# Platelet Activation Determines Angiopoietin-1 and VEGF Levels in Malaria: Implications for Their Use as Biomarkers

**DOI:** 10.1371/journal.pone.0064850

**Published:** 2013-06-03

**Authors:** Judith Brouwers, Rintis Noviyanti, Rob Fijnheer, Philip G. de Groot, Leily Trianty, Siti Mudaliana, Mark Roest, Din Syafruddin, Andre van der Ven, Quirijn de Mast

**Affiliations:** 1 Department of Clinical Chemistry and Haematology, University Medical Centre, Utrecht, The Netherlands; 2 Eijkman Institute for Molecular Biology, Jakarta, Indonesia; 3 Department of Internal Medicine, Radboud University Nijmegen Medical Centre, The Netherlands; Instituto de Higiene e Medicina Tropical, Portugal

## Abstract

**Introduction:**

The angiogenic proteins angiopoietin (Ang)-1, Ang-2 and vascular endothelial growth factor (VEGF) are regulators of endothelial inflammation and integrity. Since platelets store large amounts of Ang-1 and VEGF, measurement of circulation levels of these proteins is sensitive to platelet number, *in vivo* platelet activation and inadvertent platelet activation during blood processing. We studied plasma Ang-1, Ang-2 and VEGF levels in malaria patients, taking the necessary precautions to avoid *ex vivo* platelet activation, and related plasma levels to platelet count and the soluble platelet activation markers P-selectin and CXCL7.

**Methods:**

Plasma levels of Ang-1, Ang-2, VEGF, P-selectin and CXCL7 were measured in CTAD plasma, minimizing *ex vivo* platelet activation, in 27 patients with febrile *Plasmodium falciparum* malaria at presentation and day 2 and 5 of treatment and in 25 healthy controls.

**Results:**

Levels of Ang-1, Ang-2 and VEGF were higher at day 0 in malaria patients compared to healthy controls. Ang-2 levels, which is a marker of endothelial activation, decreased after start of antimalarial treatment. In contrast, Ang-1 and VEGF plasma levels increased and this corresponded with the increase in platelet number. Soluble P-selectin and CXCL7 levels followed the same trend as Ang-1 and VEGF levels. Plasma levels of these four proteins correlated strongly in malaria patients, but only moderately in controls.

**Conclusion:**

In contrast to previous studies, we found elevated plasma levels of Ang-1 and VEGF in patients with malaria resulting from *in vivo* platelet activation. Ang-1 release from platelets may be important to dampen the disturbing effects of Ang-2 on the endothelium. Evaluation of plasma levels of these angiogenic proteins requires close adherence to a stringent protocol to minimize *ex vivo* platelet activation.

## Introduction

The endothelium plays a central role in the pathophysiology of *P. falciparum* malaria. Erythrocytes containing mature malaria parasites adhere to the endothelium via a range of endothelial receptors in order to escape removal by the spleen. Endothelial activation is an early feature of malaria, which is likely to favor sequestration of parasitized erythrocytes [1]. Excessive activation may contribute to loss of barrier function of the endothelium and organ dysfunction. Angiogenic proteins are increasingly recognized to be central regulators of endothelial physiology. Vascular endothelial cell growth factor (VEGF) increases the expression of adhesion molecules and coagulation factors and increases vascular permeability [2], [3]. Angiopoietins are other important mediators of angiogenesis. Binding of angiopoietin (Ang)-1 to the Tie2-receptor on endothelial cells maintains endothelial integrity and reduces the effects of inflammation [4], [5]. In contrast, Ang-2 counteracts the protective Ang-1 effects and promotes vascular leakage and inflammation [6], [7]. With endothelial cell activation, cytoadherence and microvascular hypoxia as central features of malaria, it may not come as a surprise that these angiogenic proteins have been studied extensively in malaria. Circulating levels of these proteins have been determined in several studies and, collectively, these studies found reduced Ang-1 levels and elevated Ang-2 levels in patients with malaria compared to healthy controls, while data on VEGF varied across studies [8]–[10]. Additional studies suggested Ang-1 and Ang-2 to be promising biomarkers to differentiate cerebral from non-cerebral malaria [11], [12].

Both Ang-1 and VEGF are both stored in high quantities in alpha granules from platelets [13] [14] and this is especially relevant when circulating blood concentrations are measured. Ex-vivo platelet activation, which is almost inevitable unless special precautions are taken, may falsely increase blood concentrations. Platelets numbers may also influence plasma concentrations, which is especially relevant for diseases characterized by thrombocytopenia, such as malaria. Indeed, there is increasing evidence that platelets and their released proteins are important regulators of endothelial permeability and this may partly be mediated by these platelet-derived angiogenic proteins [15]. The aim of our study was to determine plasma levels of VEGF, Ang-1, Ang-2 in adult Indonesian patients with *Plasmodium falciparum* malaria from whom platelet poor plasma was obtained under special conditions to prevent ex vivo platelet activation. A second aim was to correlate plasma levels of these angiogenic proteins with circulating platelet numbers and markers of platelet activation.

## Methods

### Ethics Statement

This study received ethical clearance for the use of human subject from the Eijkman Institute Research Ethics Committee, Jakarta, Indonesia and all enrolled patients gave written informed consent.

### Study Area, Study Population, and Ethics

This study was conducted in the Rumah Sakit Karitas Hospital in Waitabula, West Sumba, East Nusa Tenggara Province, Indonesia, an area of hypo- to meso-endemic *P. falciparum* and *P.* *vivax* malaria transmission. Consecutive patients presenting to hospital with recent or current fever, clinical symptoms of malaria and a positive blood slide for *P. falciparum* were enrolled in this study following informed consent. All patients were assessed according to a predefined protocol which included a standardized history and physical examination performed by an experienced internist-infectious diseases specialist. All patients were treated with intravenous quinine and an antipyretic (paracetamol). Concurrent administration of antibiotics for severely ill patients was on the discretion of the treating physician. Venous blood for this study was collected at enrollment and on day two and five after enrollment. A group of 25 healthy young adults were recruited among local hospital staff as controls. All controls had no signs or symptoms of any illness and a negative malaria blood slide. Treatment for malaria in the past two months was an exclusion criteria for both malaria patients and controls.

### Sample Collection and Laboratory Procedures

Five ml of venous blood was collected in EDTA tubes for a full blood count and malaria slides and in CTAD tubes (Becton-Dickinson Vacutainer Systems; tubes containing citrate and the platelet stabilizing agents theophylline, adenosine, and dipyridamole) for measurement of angiogenic and platelet activation marker proteins. *In vitro* platelet activation is almost completely absent in whole blood anticoagulated with CTAD [16], [17]. CTAD blood tubes from malaria patients and controls were centrifuged within 30 minutes at 2,000 g for 10 minutes and the top fraction of the plasma was collected, carefully avoiding the buffy coat. The plasma was frozen at −20°C until shipment to a −80°C freezer at the Eijkman Institute in Jakarta. A full blood count was determined by a standard hematology analyzer (Arcus, Diatron, Vienna, Austria). Thick and thin blood smears were stained with Giemsa, and the number of parasites was quantified against 200 white blood cells. Parasite density was calculated using the patient’s white blood cell count. Plasma Ang-1, Ang-2 and VEGF levels were measured by quantitative sandwich enzyme immunoassay technique according to the instructions of the manufacturer (Quantikine, R&D systems, Minneapolis, USA) at the Eijkman Institute. The platelet activation markers P-selectin and CXCL7 (beta-thromboglobulin) were measured in the Department of Clinical Chemistry and Haematology of the University Medical Center Utrecht as described in detail earlier [18].

### Statistical Analysis

Data are presented as median followed by interquartile range in parentheses unless otherwise stated. Within the group of malaria patients, the Freidman test with post-tests was used to compare laboratory parameters on the three time points.Mann-Whitney U test was used for comparisons with the controls. Relationships between laboratory parameters were assessed using Spearman correlation coefficient. All analyses were performed with GraphPad version 5.0.

## Results and Discussion

A total number of 27 patients with *P. falciparum* malaria and 25 controls were included. Demographic and clinical characteristics are shown in table 1. Compared to the controls, malaria patients were younger and more often male. Both groups shared the same genetic background. None of the patients and controls had been treated for malaria in the past two months or had used medication in the week before enrollment. Moreover, none had diabetes mellitus or had suffered from a cardiovascular event or tuberculosis in the past. Malaria patients had a significantly lower platelet count and a lower hemoglobin level. According to WHO-criteria, two malaria patients were classified as suffering from cerebral malaria and four as severe malarial anemia.

**Table 1:** Characteristics of malaria patients and controls.

	*P. falciparum* malaria	Healthy controls	P value
Number, n	27	25	
Age, years	13 (6–26)	24 (22–29)	0.001
Female, n (%)	9 (33)	19 (76)	0.02
Native Sumbanese, n (%)	26 (96)	25 (100)	ns
Smoking, n (%)	6 (22)	4 (16)	ns
Days illness, days	3 (3–5)	NA	
Last malaria treatment, months	24 (11.5–60)	48 (24–60)	0.04
Parasite density, parasites/mL	71,203 (22,055–167,560)	0 (0–0)	
Platelet count, 109/L	70 (54–112)	360 (266–437)	<0.001
Platelet count <150×109/L, %	21 (78)	0 (0)	
Mean platelet volume, fl	7.2 (6.5–8.0)	6.8 (5.9–7.8)	ns
Hemoglobin, g/dL	10.7 (9.4–12.8)	13.3 (12.4–14.1)	<0.001
White blood cell count, 109/L	6.7 (5.4–9.9)	6.9 (6.1–7.3)	ns

Shown are medians with interquartile range unless otherwise specified.

ns, not significant (P>0.05); NA, not applicable.

**Figure 1:**
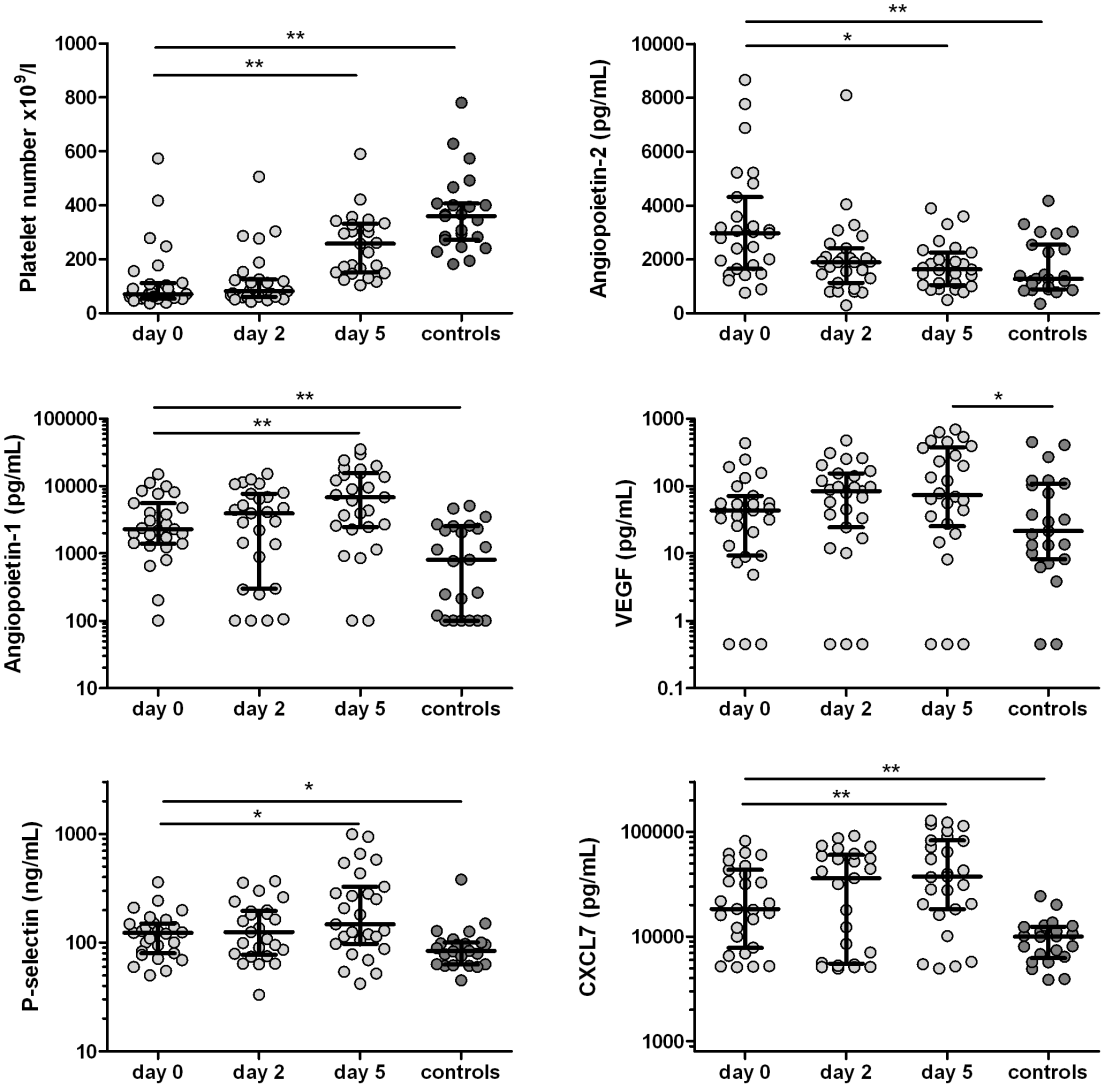
Platelet number and plasma levels of angiogenic and platelet activation markers. Levels of angiopoietin-2, angiopoietin-1, vascular endothelial growth factor (VEGF), soluble P-selectin and CXCL7 were determined in CTAD plasma in 27 patients with febrile *P. falciparum* malaria at the start of malaria treatment (day 0), day 2 and day 5 and in 25 healthy controls. Data are presented in a scatter dot plot with median and interquartile range. Differences between malaria patients in time were assessed by the Friedman’s test; differences with controls with the Mann-Whitney test. **denotes p<0.01 and *p<0.05.

Figure 1 shows the course of platelet numbers, together with the course of the angiogenic proteins Ang-1, Ang-2 and VEGF and of the platelet activation markers P-selectin and CXCL7. As expected, platelet numbers rose after start of antimalarial treatment on day 0. Ang-2 is stored in Weibel-Palade bodies in endothelial cells and is therefore regarded as a marker of endothelial cell activation. Plasma levels of Ang-2 were elevated at day 0 and decreased upon start of antimalarial treatment to levels comparable to those in controls. In contrast, Ang-1 and VEGF plasma levels were also higher at day 0 compared to levels in controls, but start of antimalarial treatment resulted in a further increase in these proteins. The ratio of Ang-2/Ang-1 in the day 0 sample of patients and controls was similar (1.4 vs. 1.3; p = 0.18). After start of antimalarial treatment, the Ang-2/Ang-1 ratio declined to 0.5 at day 2 and 0.3 at day 5. the six patients with severe malaria had a higher median Ang-2/Ang-1 ratio at day 0 (1.5 vs. 0.8) and day 2 (4.7 vs. 0.5), although these differences did not reach statistical significance.

The time course of Ang-1 and VEGF resembled the course of the platelet activation markers P-selectin and CXCL7. Correlation analysis, presented in table 2, showed that both Ang-1 and VEGF levels in malaria patients were strongly correlated with P-selectin and especially CXCL7 levels on the three time points. While P-selectin is released by both activated platelets and endothelial cells, CXCL7 is exclusively derived from platelets [19]. There was no significant correlation with either Ang-2, platelets counts or WBC counts. Moreover, in controls, correlations between Ang-1 and VEGF and markers of platelet activation were only weak. These findings suggest that platelet activation is one of the main determinants of plasma levels of Ang-1 and VEGF in malaria patients, but not in healthy controls. The fact that VEGF is made by many different cell types, and its production is upregulated in hypoxic tissues may explain the weaker correlation of VEGF than Ang-1 with other platelet activation markers. We have previously reported that malaria is associated with a normal thrombopoietic activity [20]. Ongoing peripheral clearance of degranulating platelets may explain why plasma levels of these platelet-stored proteins were increased despite the low circulating platelet counts. The rise in circulating platelet counts following start of antimalarial treatment together with ongoing platelet degranulation may also explain the further increase in plasma levels of these proteins, although no significant correlation of platelets numbers with Ang-1 and VEGF plasma levels was found. Age did not correlate with protein levels of any of the measured proteins (data not shown), suggesting that age difference between malaria patients and controls did not influence our findings.

**Table 2:** Spearman correlation of angiopoietin-1 and VEGF with other parameters.

	Ang-1				VEGF			
	day 0	day 2	day 5	controls	day 0	day 2	day 5	controls
VEGF	0.54‡	0.82‡	0.84‡	−.09				
Soluble P-selectin	0.64‡	0.87‡	0.84‡	0.55‡	0.48†	0.60‡	0.86‡	0.11
CXCL7	0.81‡	0.90‡	0.95‡	0.45†	0.58‡	0.66‡	0.89‡	−.28
Ang-2	0.07	−.16	0.01	−.03	−.16	−.08	0.31	−.03
Platelet counts	−.14	0.01	0.17	0.17	−.08	0.09	0.06	0.06

† p<0.05.

‡ <0.01.

These findings are in contrast to previous studies which showed that patients with malaria had lower Ang-1 levels than healthy controls [8], [9]. Within the group of malaria patients, those with the most severe illness also had lower levels [11], [12]. Data on VEGF in malaria patients varied more across studies with both higher and lower levels reported [10], [21], [22]. Importantly, to our knowledge, in none of the studies, CTAD or citrate was used as anticoagulant. As described above, when measuring platelet-stored proteins like Ang-1 and VEGF, selecting the right anticoagulant and blood collection and processing techniques is of paramount importance. If the goal is to assess physiological plasma levels, EDTA or heparin plasma are less suitable as these anticoagulants are known to cause various degrees of *in vitro* platelet activation [23], [24], [25]. While sodium citrate as anticoagulant already results in a major reduction in *ex vivo* platelet activation, CTAD tubes almost completely prevent in vitro platelet activation and several authors have already recommended using CTAD when platelet-stored angiogenesis proteins are being measured [17], [25]–[28]. When these special measures are not taken, the circulating platelet number may have a strong impact on measured concentrations of platelet-stored proteins. Patients with malaria frequently have thrombocytopenia and platelet numbers have been shown to inversely correlate with severity of illness. Thus, we speculate that the lower circulating platelet numbers together with variable degrees of *ex vivo* platelet activation was responsible for the lower Ang-1 and VEGF concentrations in previous studies. Serum concentrations of platelet-stored angiogenic proteins may also have prognostic value. Generation of serum results in complete *in vitro* platelet activation and serum concentrations will largely reflect the total platelet content of angiogenic proteins. Possible drawbacks of using serum for this objective is that clotting processes may not release all platelet-stored angiogenic growth factors into the serum [29] and that serum concentrations may have no additional prognostic value over platelet count, as serum concentrations will heavily depend on platelet counts.

We only used a single centrifugation step to generate platelet poor plasma in our study, while a double centrifugation step is often advised. We assumed that only under special circumstances single centrifugation does not result in near complete removal of platelets from the top layer of the plasma. We tested this assumption by centrifuging venous blood in CTAD tubes from five volunteers using the same procedures as in our study and we found that a singly centrifugation step resulted in no detectable platelets in the plasma in four volunteers and a negligible platelet number (2×10^9^/L) in the remaining volunteer.

Several groups of researchers have investigated the utility of angiopoietins and VEGF as biomarkers to identify patients with severe malaria. Due to its limited sample size, our study did not allow to explore the role of these angiogenic proteins in malaria. In general, we strongly recommend that a similar protocol for measurement of platelet-stored angiogenic proteins is used in future biomarker studies, including use of CTAD plasma and consideration of circulating platelet numbers. These precautions do not apply to Ang-2, which is derived from endothelial cells and of which plasma levels are not affected by platelet activation.

What are the implications of these findings for the pathophysiology of malaria? Increasing evidence supports a central role for platelets in protecting the vasculature during inflammation [15], [30], [31]. The exact mechanisms are still unclear, but soluble factors released from platelets may play a role [15]. The angiopoietin-Tie-2 system has evolved as a central regulator of the activation status and permeability of the vascular lining. An imbalance in the pro-permeability and pro-inflammatory effects of Ang-2 and VEGF and the anti-permeability effects of Ang-1, together with the disrupting effects of pro-inflammatory cytokines on vascular integrity, may contribute to transient plasma leakage. Our current data suggest that there is indeed a distortion of this balance. Ang-1 release from platelets may especially be important in these circumstances to dampen the disturbing effects of Ang-2 on the endothelium. Since both platelet and endothelial activation are common in many infectious diseases, the changes in angiogenic proteins observed in our study may not be specific to malaria.

In conclusion, we show that platelet activation is an important determinant of circulating Ang-1 and VEGF levels in malaria. Optimal biomarkers for malaria severity would rely on inexpensive point-of-care devices using whole blood. The fact that assays measuring these platelet-stored angiogenic proteins require special precautions to avoid inadvertent platelet activation during blood processing and are prone to artifacts limits widespread use of these proteins as possible biomarkers.
